# Roles of Inflammasomes in Inflammatory Responses and Human Diseases

**DOI:** 10.3390/ijms26115016

**Published:** 2025-05-23

**Authors:** Young-Su Yi

**Affiliations:** Department of Life Sciences, Kyonggi University, Suwon 16227, Republic of Korea; ysyi@kgu.ac.kr; Tel.: +82-31-249-9644

Inflammation is an innate immune defense that protects our bodily tissues from pathogens and cellular dangers [1,2]; however, repeated and prolonged inflammation, known as chronic inflammation, has been considered a key risk factor for a variety of human diseases, including inflammatory, autoimmune, and infectious diseases, and even cancers [3,4,5]. An inflammatory response is initiated when the pathogen recognition receptors (PRRs) of the inflammatory cells recognize the pathogen-associated molecular patterns (PAMPs) or danger-associated molecular patterns (DAMPs), which results in the activation of multiple inflammatory signaling pathways [2,6,7,8]. An inflammatory response consists of two successive steps: priming and triggering. Priming is the step of preparing inflammatory responses by upregulating inflammatory genes, while triggering is the step of activating and boosting inflammatory responses by activating inflammasomes and the intracellular multiprotein complexes responsible for the stimulation of inflammatory responses [2,6,7,8]. Therefore, inflammasome activation has been regarded as a key determinant of the induction of inflammatory responses and the progression of various human diseases, whilst the inhibition of inflammatory responses via the selective targeting of inflammasomes has been suggested as a promising strategy to develop novel therapeutics for the treatment of these human diseases [9,10,11]. However, the roles of inflammasomes and their dysregulation during inflammatory responses and human diseases are still largely unknown and remain to be investigated.

This Special Issue welcomes studies exploring but not limited to the “Roles of Inflammasomes in Inflammatory Responses and Human Diseases”, identifying and validating the novel targets regulating inflammasome functions, evaluating anti-inflammatory effects by targeting inflammasomes, and developing potential inflammasome-targeted therapeutics.

The review article by Yi highlights the regulatory roles of the caspase-11 noncanonical inflammasome in a range of rheumatic diseases. This review offers a comprehensive overview of the recent studies highlighting the involvement of human caspase-4 and murine caspase-11 noncanonical inflammasomes in the development and progression of various rheumatic conditions, including rheumatoid arthritis (RA), infectious arthritis (IR), gouty arthritis (GA), osteoarthritis (OA), systemic lupus erythematosus (SLE), psoriatic arthritis (PA), ankylosing spondylitis (AS), and Sjögren’s syndrome (SjS) across cellular models, animal studies, and human patients. Additionally, the review presents novel perspectives on therapeutic strategies aimed at preventing and treating rheumatic diseases and their related conditions by targeting noncanonical inflammasomes.

The cross-sectional study by Chuang et al. evaluated the associations between weight status, autonomic function, and systemic inflammation in a group of 55 children diagnosed with obstructive sleep apnea (OSA). Using multiple linear regression analysis, the study found that the apnea−hypopnea index was significantly linked to body mass index (BMI), the standard deviation of successive differences between normal-to-normal intervals during N3 sleep, and the proportion of these interval pairs differing by more than 50 milliseconds during REM sleep. Furthermore, a moderated mediation model revealed that IL-1 receptor antagonist levels mediated the relationship between the BMI and IL-6 levels, with sympathovagal balance during N3 sleep and minimum oxygen saturation acting as moderators. These findings underscore the intricate interplay between BMI, sleep-related physiological markers, and inflammation in pediatric OSA, highlighting the critical role of weight management in this population.

The research article by Huang et al. examined the intricate associations between individual donor characteristics and cytokine responses during H3N2 influenza A virus (IAV) infection. Following infection, several key cytokines were upregulated. Notably, a prior history of lung cancer did not affect the acute immune response. However, cigarette smoking and elevated programmed cell death protein 1 (PD-L1) expression on macrophages were linked to increased IL-2 levels. In contrast, age was associated with reduced IL-4 levels, and diabetes mellitus was linked to lower IL-6 levels. Additionally, both diabetes and PD-1 expression on CD3+/CD4+ T cells were negatively correlated with TNF-α levels, while a higher body mass index was associated with decreased IFN-γ production. The study also identified TLR2 expression as a key mediator of both innate and adaptive immune responses. These results emphasize the complex interactions between individual physiological factors and immune dynamics during influenza infection, highlighting the need for personalized approaches in managing and preventing IAV.

The research article by Catalina et al. evaluated the activation of inflammatory and apoptotic pathways in the livers of lactating dams following exposure to 1,2-cyclohexane dicarboxylic acid diisononyl ester (DINCH), as well as the potential effects on their offspring at postnatal day 6 (PND6). Exposure to a low dose of DINCH led to the increased production of pro-inflammatory cytokines, elevated oxidative stress, higher caspase-3 levels, and the reduced expression of anti-apoptotic proteins in both lactating dams and PND6 offspring. These results indicate that even low-dose, continuous DINCH exposure can disrupt the balance of inflammatory and oxidative stress responses, causing liver damage in lactating mothers and exerting early-life effects on their offspring.

The research article by D’Amico et al. investigated whether the chronic activation of the NLRP3 inflammasome in individuals with obesity differs between critical COVID-19 cases and other severe chronic conditions. In obese patients with critical COVID-19, there was a significant upregulation of ASC and caspase-1 expression, while the levels of IL-8, TNF-α, NF-κB, NLRP3, IL-1β, and gasdermin-D did not differ significantly. In contrast, obese individuals who died from non-COVID-19 causes exhibited elevated levels of IL-6, IL-18, caspase-9, and HIF. These findings suggest that COVID-19 in obese patients may drive NLRP3 inflammasome activation, potentially triggering pyroptotic cell death via caspase-1. In contrast, in obese patients without COVID-19, cell death appears to be more closely linked to necroptosis, mediated by caspase-9.

The research article by Berk et al. investigated the impact of inhibiting phosphodiesterase 10A (PDE10A) on inflammasome-mediated inflammation, using two inhibitors—MP-10 and TP-10—in both macrophage cultures and animal models of sepsis and traumatic nerve injury. PDE10A inhibition suppress inflammasome activation by blocking ASC speck formation and reducing the levels of key components involved in pyroptosis, such as cleaved caspase-1, gasdermin D, and IL-1β. In the sepsis model, MP-10 significantly lowered inflammation, decreased the plasma IL-1β levels, alleviated thrombocytopenia, and improved indicators of organ damage. In the nerve injury model, PDE10A inhibition promoted motor function recovery and reduced the expression of genes associated with muscle atrophy. These findings suggest that PDE10A may be a promising therapeutic target for treating inflammatory and neuromuscular disorders.

This Special Issue emphasizes the multifaceted roles of inflammasomes in inflammatory processes and human diseases, along with the molecular mechanisms involved. We hope that this Special Issue helps to enhance the current knowledge on and understanding of inflammasomes in these contexts and offers valuable insights that can guide future research on their emerging functions. We further hope that this Special Issue contributes to the development of novel therapeutic strategies for preventing and treating a wide range of human diseases by targeting inflammasome-driven inflammation.
